# Analysis of vibratory mode changes in symmetric and asymmetric activation of the canine larynx

**DOI:** 10.1371/journal.pone.0266910

**Published:** 2022-04-14

**Authors:** Patrick Schlegel, David A. Berry, Dinesh K. Chhetri

**Affiliations:** Department of Head & Neck Surgery, David Geffen School of Medicine, University of California Los Angeles (UCLA), Los Angeles, CA, United States of America; University of Colorado School of Medicine, UNITED STATES

## Abstract

Investigations of neuromuscular control of voice production have primarily focused on the roles of muscle activation levels, posture, and stiffness at phonation onset. However, little work has been done investigating the stability of the phonation process in regards to spontaneous changes in vibratory mode of vocal fold oscillation as a function of neuromuscular activation. We evaluated 320 phonatory conditions representing combinations of superior and recurrent laryngeal nerve (SLN and RLN) activations in an *in vivo* canine model of phonation. At each combination of neuromuscular input, airflow was increased linearly to reach phonation onset and beyond from 300 to 1400 mL/s. High-speed video and acoustic data were recorded during phonation, and spectrograms and glottal-area-based parameters were calculated. Vibratory mode changes were detected based on sudden increases or drops of local fundamental frequency. Mode changes occurred only when SLNs were concurrently stimulated and were more frequent for higher, less asymmetric RLN stimulation. A slight increase in amplitude and cycle length perturbation usually preceded the changes in the vibratory mode. However, no inherent differences between signals with mode changes and signals without were found.

## I. Introduction

Various works have been published investigating every aspect of voice research; ranging from simulation of fluid mechanics and vibration in the context of voice analysis [1–3] to analyzing development, structure and composition of the larynx, the vocal folds and other parts of the voice apparatus [4–6]. However, there are still topics remaining that have not received as much attention, one of these topics being phonatory stability and spontaneous mode changes.

The process of phonation starts with the lungs generating a pressure and pushing air through the trachea upwards. As this air passes the vocal folds located in the larynx, it sets them in motion. The high frequency vibration of the vocal folds subdivides the airstream in a series of flow pulses, generating the fundamental frequency of phonation. This sound is then further modulated using vocal tract, tongue and lips resulting in audible speech [6, 7].

In the larynx, vocal folds tension and posture are adjusted using different muscles activated by certain laryngeal nerves. For this study, two types of laryngeal nerves are of interest: The recurrent laryngeal nerve (RLN), activating all the laryngeal intrinsic adductor muscles that close the glottal gap and stiffen the body layer, and the superior laryngeal nerve (SLN), activating the cricothyroid (CT) muscles that elongate and tense the vocal folds. Both nerves innervate the larynx on the left and right side resulting in two branches that can be stimulated independently [8].

The phonatory stability of vocal fold oscillation has mostly been investigated in regard of voluntary changes in vibratory mode by adjusting muscles, as they occur for instance during professional singing when switching between registers [9–12]. However, there are also spontaneous changes in vocal fold vibratory mode that can lead to frequency drops or jumps. Certain patterns of muscle activation also may lead to a more stable or more instable vibration [13].

Vibratory function and changes in voice and pitch are often accessed indirectly via methods such as radiography and inverse filtering [14, 15] or, more recently, 3D tomography [16]. To work around the limitations of indirect measurement and to allow a precisely defined “input” in regards of muscle activation, an *in vivo* animal phonation model can be used. In this way, vocal fold vibration patterns can be recorded, and laryngeal nerves can be stimulated directly. This approach was developed and successfully implemented previously by Chhetri at al. [17–19] for various nerve stimulation experiments and is described in the Method section.

Synchronous acoustic and high-speed video (HSV) recordings of the in vivo canine larynx provide a comprehensive data base for analysis. From the HSV recordings, the glottal area over time i.e. the “Glottal area waveform” (GAW) can be extracted. The GAW displays the glottal area between the vocal folds for each video frame [20] and can be used to extract basic oscillation behavior (like opening and closing times) and parameters. It therefore describes the pattern of vocal fold oscillation that produces the resulting acoustic signal.

For this study three previous works are of particular interest: In 2016 Chhetri and Park investigated the influence of various combinations of thyroarytenoid, lateral cricoarytenoid/interarytenoid and CT activation on the acoustic output in an in vivo canine model during phonation [21]. As a side result of their study, they found decreased stability in phonation at high CT activation leading to vibratory mode changes. In 2018 Zhang investigated vocal instabilities using a three-dimensional body-cover-phonation model. His model predicted that a tighter approximation and lower transverse stiffness of vocal folds would lead more often to irregular vibration [13]. Berry et al. (1996) investigated spontaneous shifts in the vibratory mode of the vocal folds in excised canine larynx experiments [22]. That is, as a function of asymmetrical forces (both in vocal fold elongation and arytenoid adduction) and subglottal pressure, spontaneous jumps between a variety of modes were recorded and analyzed, among others, jumps from chest-like vibrations to falsetto-like vibrations, from chest-like vibrations to vocal-fry like vibrations, from chest-like vibrations to irregular or chaotic vibrations, and from single vocal-fold vibrations to chest-like vibrations.

The current study proposes to significantly build upon Berry et al. (1996) by using an intact neurological model of the larynx (the *in vivo* canine laryngeal model), and by performing a systematic, comprehensive investigation of asymmetrical neurological conditions with regard to both vocal fold elongation and vocal fold adduction. To allow for a more in-depth analysis of spontaneous mode changes and factors that may contribute to such jumps or drops in F0 happening, in this study we use a combination of acoustic and HSV based data evaluation. Stimulation of SLNs and RLNs over 320 different combinations of activation ensures a broad but not excessive spectrum of phonation configurations. To help ensure the reproducibility of our rather complex approach, we publish all code that was used for outlier removal, parameter calculation and mode change detection in the supporting information.

The aims of this investigation were as follows: (a) Investigate mode changes as a function of RLN and SLN activation, (b) explore acoustic and vibratory events just before, during and after a mode change, and (c) evaluate parameters that may differentiate signals with and without accompanying mode changes.

## II. Materials and methods

This study was carried out in strict accordance with the recommendations in the Guide for the Care and Use of Laboratory Animals of the National Institutes of Health. The Animal Research Committee (ARC) of the University of California, Los Angeles, approved this study (Protocol Number: ARC-2010-021). For this study data derived from a mongrel canine, sourced from Oak Hill Genetics (Merced, California, USA) was used. The canine received a physical exam within one day of arrival to UCLA and was monitored following the HERSLAW method for food and water intake, urine and feces production and general appearance. It was housed in compliance with the Guide for the Care and use of Animals and the Animal Welfare Act until surgery and received a diet of canned food, lamb and rice formula. Environmental enrichment was provided in accordance with the institutional canine enrichment program, including toys, human interaction and increased space.

The canine model is necessary and suitable for this study as it represents the human larynx most closely in larynx size, neuromuscular anatomy, and histopathology of the vocal folds. Data from a previously performed experiment was analyzed [19]. While the previous report focused on events at phonation onset, this analysis used a larger dataset looking for mode changes beyond phonation onset.

### A. Preparation for in vivo phonation

One mongrel canine was used for in vivo RLN and SLN stimulation. Preparation and surgical exposure of the laryngeal nerves has been described in detail before [18, 19] and can be summarized as follows: The canine larynx was exteriorized in the neck, and laryngeal nerves were dissected. For intraoperative ventilation, a tracheostomy was placed. Tripolar cuff electrodes were placed around the laryngeal nerves for graded neuromuscular activation. To prevent cross stimulation, internal SLN branches and nerve branches to the posterior cricoarytenoid muscles (PCA) as well as Galen’s anastomosis were severed bilaterally. The internal branch of the superior laryngeal nerve and the Galen’s anastomosis do not contain any motor nerves that we know of, but we divide them just as a precaution. The PCA muscle nerve branch takes off as the first branch of the recurrent laryngeal nerve (RLN) and we divide this branch so we can focus just on the laryngeal adductor muscles. Furthermore, if the PCA branch is not divided there can be opposing forces to laryngeal adduction and occasionally even abduction. Sectioning of these nerves does not change stimulability of the tested muscles. To achieve phonation, rostral airflow was provided using a subglottal tube attached to the trachea at approximately tracheal rings two through four. Airflow rate was increased linearly using an airflow-controller (MCS Series Mass Flow Controller; Alicat Scientific, Tucson, AZ) from 300 ml/s at onset of neuromuscular stimulation to 1,400 ml/s at the end of stimulation (1.5 s). Airflow at glottis level was heated and humidified to 37.5° and 100% using a heated humidifier (HumiCare 200; Gruendler Medical, Freudenstadt, Germany).

### B. Asymmetric nerve stimulation protocol

Before the start of the actual stimulation protocol threshold and maximum nerve stimulation levels were determined. At threshold nerve stimulation, there is just hint of muscle activation and at maximum stimulation the vocal folds reach maximum excursion. Based on these limits eight equidistant levels of stimulation for RLNs and five levels of stimulation for SLNs (both including no stimulation) were applied in various RLN/SLN combinations.

The nerve stimulation protocol consisted of five sets. For each set bilateral symmetric SLN stimulation was kept constant (five levels, including zero activation condition), while the left and right RLN stimulation was varied in combinations to achieve symmetric and asymmetric activation (eight levels, including zero stimulation). Thus, each stimulation set consisted of 64 neuromuscular activation conditions and a total 320 combinations of SLN and RLN activation were tested, as summarized in Table 1. The duration of neuromuscular activation was 1.5 s and 3.5 s recovery time was allowed prior to the next activation condition. Stimulation was achieved using 0.1 ms unipolar cathodic pules at a 100 Hz pulse repetition rate.

**Table 1:** Neuromuscular conditions tested.

	SLN activation	RLN activation
Set 1	Symmetric, level 0	64 combinations, level 0–7 (left and right)
Set 2	Symmetric, level 1	64 combinations, level 0–7 (left and right)
Set 3	Symmetric, level 2	64 combinations, level 0–7 (left and right)
Set 4	Symmetric, level 3	64 combinations, level 0–7 (left and right)
Set 5	Symmetric, level 4	64 combinations, level 0–7 (left and right)

### C. Data recording

Three hundred twenty high-speed videos with synchronous audio for each stimulation combination were collected. High-speed video-data of the vocal fold oscillations was recorded using a Phantom v210 high-speed camera (Vision Research Inc., Wayne, NJ) with a sampling rate of 3000 frames per second at a fixed distance. Acoustic data was recorded using a probe tube microphone (Model 4128; Bruel & Kjaer North America, Norcross, GA) with a sampling rate of 50,000 Hz. The microphone was mounted at the inner wall of the subglottic inflow tube, allowing for low-noise recording of canine phonation.

Phonation onset was manually determined for each video using the high-speed video recordings and acoustic signals. To exclude very early onset and potential early offset from analysis, the first and last 10 ms (30 frames) of the phonation segments were discarded. Further, all data sets that did not achieve at least 0.25 s (750 frames) of continuous phonation following onset were excluded from analysis, resulting in 183 remaining data sets.

### D. Segmentation

**Fig 1:**
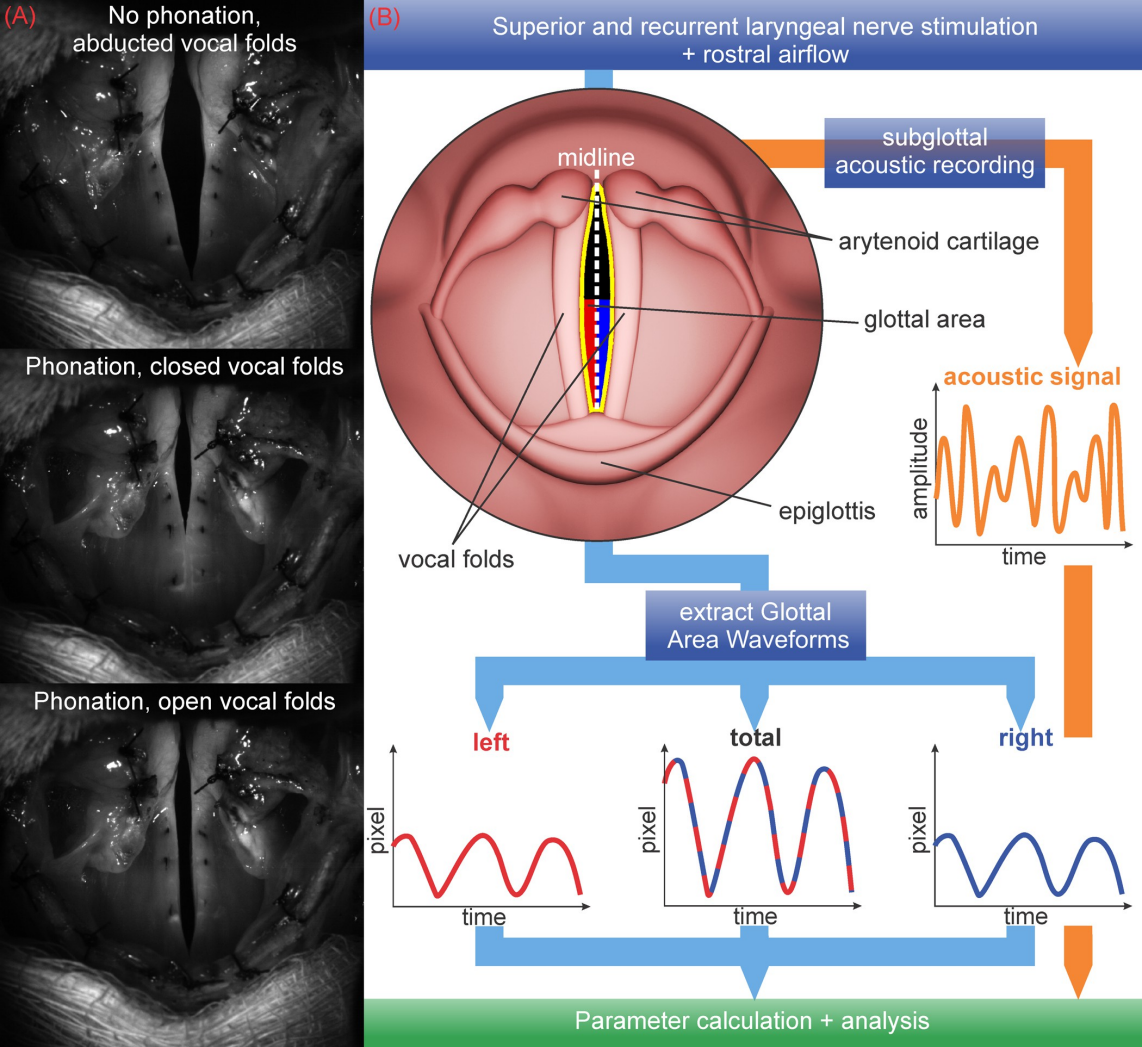
(A) Vocal folds in abducted state and during phonation (B) Glottal area segmentation and GAW extraction.

For all 183 remaining high-speed videos, the glottal area between the vocal folds was segmented using a custom version of the software “Glottis Analysis Tools 2020” (GAT), featuring a static midline. A detailed description of this tool can be found in Kist et al. [23] and the segmentation process is explained in detail in Schlegel et al. [24]. Most of the constantly open posterior cartilaginous section of the glottis was excluded from segmentation, as only the changing membranous glottal area was of interest in the subsequent analysis. For each video a static midline was drawn and total left and right GAWs were extracted (e.g., left and right halves of the glottal area separated by the midline). The sections of the acoustic signal that were synchronous with the extracted GAWs were derived as well. Different states of the vocal folds (abducted and maximum open and closed during phonation) are shown in Fig 1(A). A pictorial summary of the segmentation process and data extraction is given in Fig 1(B).

### E. Parameter analysis

Extracted GAW- and acoustic data was exported to Matlab (version 9.9.0.1538559 (R2020b) Update 3). Based on the total GAW, local maximum based cycles were detected using the “findpeaks” function. As a minimum distance between peaks (’MinPeakDistance’), 3 frames (1 ms) were chosen. The minimum height (’MinPeakProminence’) was set as 5% of the maximum dynamic range (highest distance between a local peak and a close local minimum (within 12,5 ms)). This relative value was chosen to accommodate the differences in maximum amplitude between signals and to factor in the general increase of acoustic amplitude over time. For some of the high-speed videos, a small number of consecutive frames were damaged (always below 20). Cycles that contained damaged sections were excluded from analysis. Further, outlier-Cycles were excluded by comparing each cycle length with the local median cycle length (the exact algorithm is included in S1 File). Cycles that were extremely short / long in comparison to the local median length were excluded.

To investigate basic properties of the oscillation process over time, seven different cycle-based parameters were calculated on the GAWs. Names, units, sources, abbreviations and a short explanation for all these parameters are given in Table 2. All of these parameters were implemented in Matlab based on the respective implementations in GAT [23]. This was necessary, as the data could not be processed using GAT due to the damaged sections and the changed cycle detection.

**Table 2:** Name, source, unit, abbreviation and a short explanation of all calculated parameters.

Name, unit and source	Abbreviation	Explanation
Local Fundamental Frequency (Hz)	LF0	Inverse Cycle duration i.e. fundamental frequency of cycles
Dynamic Range (Pixel)	DR	Maximum—minimum of Cycle
Phase Asymmetry (a.u.) [25]	PHA	Relative difference between local maxima of left and right partial GAW
Amplitude Periodicity (a.u.) [26]	AP	Relative difference between dynamic ranges of neighboring cycles
Time Periodicity (a.u.) [26]	TP	Relative difference between durations of neighboring cycles
Open Duration (ms)	OD	Duration of glottal opening time (between cycle minimum and end)
Closing Duration (ms)	CD	Duration of glottal closing time (between cycle beginning and minimum)

Mode changes were detected based on Local Fundamental Frequency (LF0). For mode change detection, cycle-based LF0 values were filtered using a moving median filter (movmean function, ten-point mean values) to better reflect the general LF0 trend. Respectively, first and last five cycles were excluded. A change in F0 was considered a mode change if averaged LF0 increased or decreased by 50 Hz or more within ten cycles over a range of at least five consecutive cycles. The 50 Hz threshold was chosen as it was high enough to be clearly audible and lead to a visible change in the signal but also low enough to not exclude smaller potential mode change events. Changes extremely early or late in the signal were not detected as it could not be ensured that the change was not only a side effect of onset / offset. All scripts that were used for cycle detection, parameter calculation and mode change detection are included in the supporting information (S1 and S2 Files).

## III. Results

The number of neuromuscular activation conditions included in analysis varied dependent on SLN level. Most conditions were included for dataset with no SLN stimulation (53 of 64) and least for the highest SLN stimulation (24 of 64), since phonation onset occurred less often with increasing SLN activation level at low RLN activation levels due to larger glottal gap and increased stiffness of the glottis.

**Fig 2:**
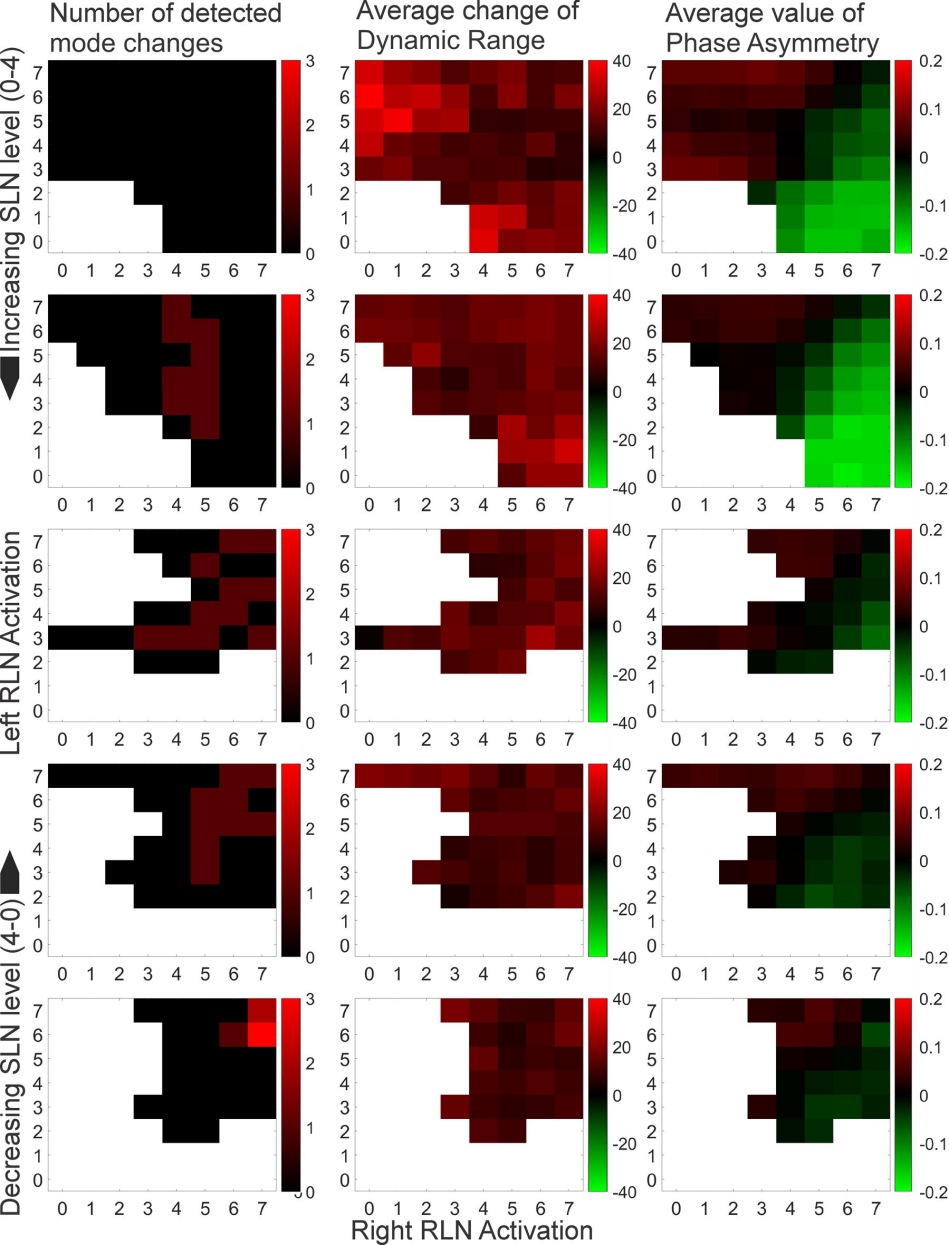
Detected mode changes, change of DR and symmetry relative to SLN and RLN activation. Blank (white) data points reflect neuromuscular conditions where phonation onset did not occur. In the first column red shades reflect the number of detected mode changes; in the second column red shades reflect an increase in dynamic range over time; in the third column red shades reflect a left sided phase lead and green shades a right sided one.

For all activation combinations oscillation amplitude increased with increasing flow. Asymmetric stimulation was also reflected well by asymmetric Glottis oscillation (vibratory phase asymmetry). F0-based mode changes occurred only in datasets with present SLN stimulation and were slightly (but not distinctly) more frequent for higher flow rates and more symmetric RLN activation conditions. In almost all cases except for the highest SLN stimulation either none or a single mode change occurred per signal. An overview of these findings is given in Table 3 and Fig 2.

**Table 3:** Observed F0 mode changes relative to SLN activation.

SLN level	Included neuromuscular conditions	Signals with mode changes	Max. changes per signal	Average Flow on mode change
0	53	0	-	-
1	45	9	1	904
2	28	11	1	1197
3	33	9	1	1126
4	24	3	3	1191*

* only signals with single mode changes were included i.e. only one signal for this SLN level

### A. Parameter analysis

**Fig 3:**
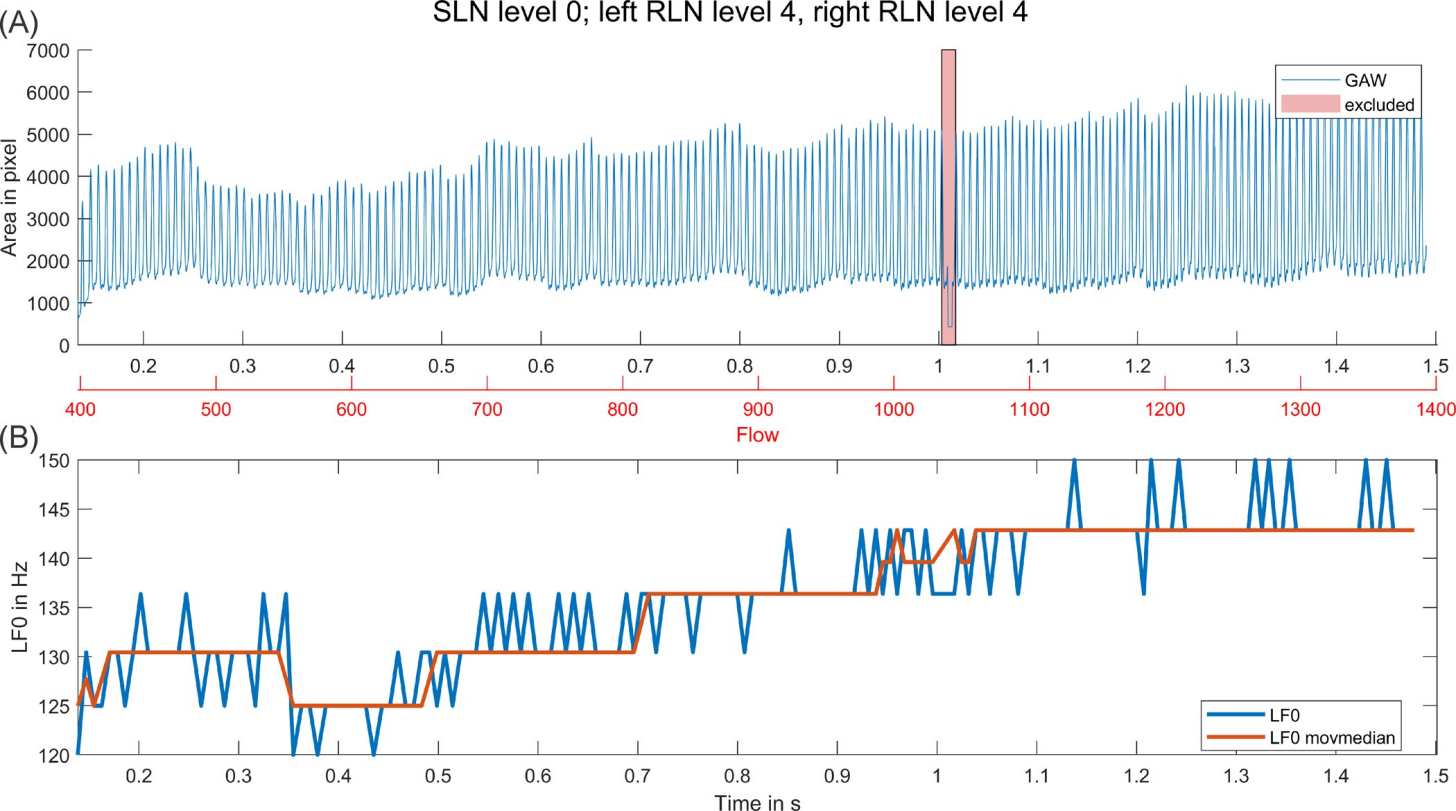
Example for small LF0 increase over time for no SLN stimulation. (A) GAW over period of stimulation with increasing flow, excluded section due to camera malfunction is marked. (B) LF0 and median LF0 (10 cycles) over same period.

**Fig 4:**
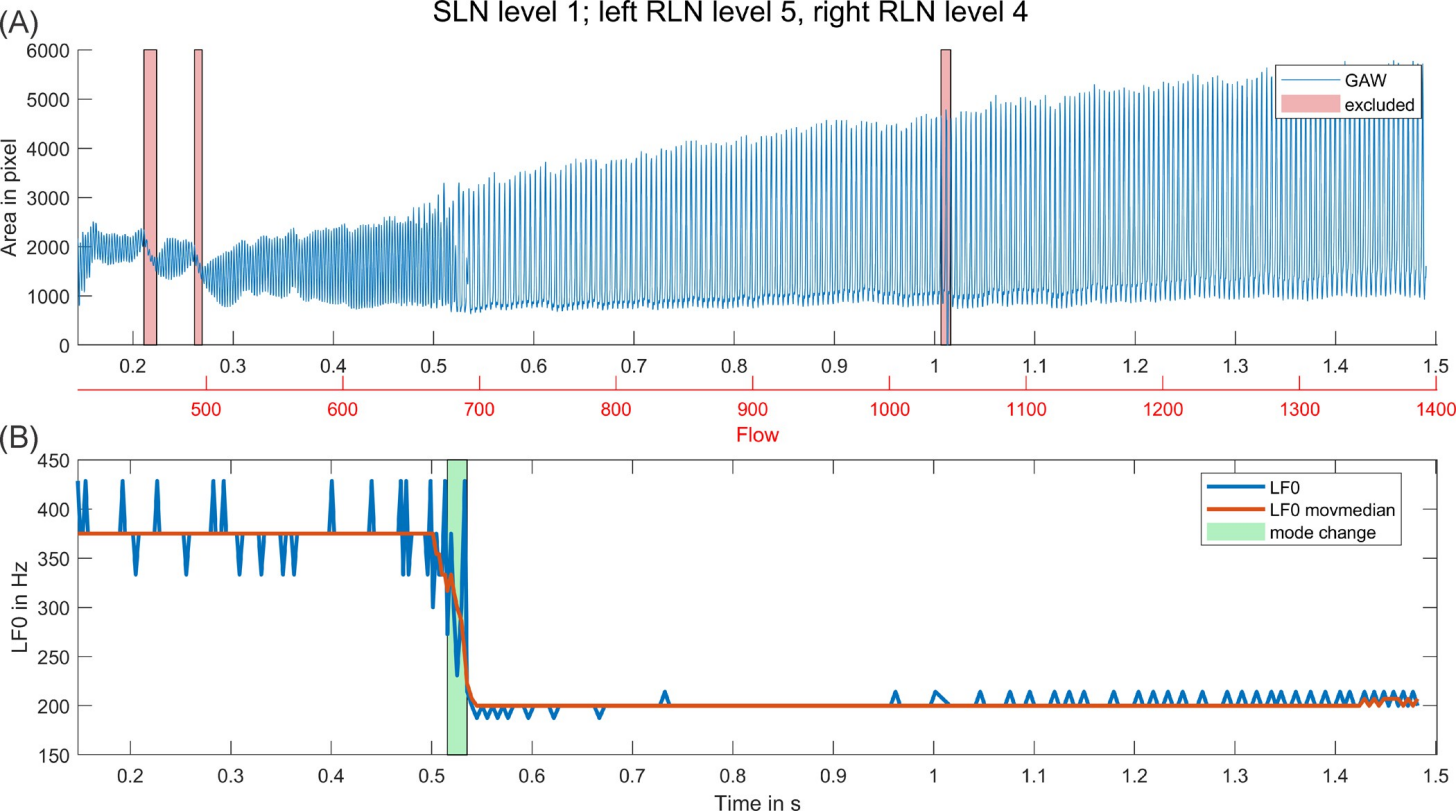
Example for a typical mode change. (A) GAW over period of stimulation with increasing flow, excluded sections due to bad cycle detection / camera malfunction are marked. (B) LF0 and median LF0 (10 cycles) over same period with marked mode change at 0.52 s.

In cases of no SLN activation, LF0 appeared to be less stable over time and often increased with increasing flow. In cases of SLN activation, LF0 was higher in general and subjectively more stable. However, if a mode change occurred this almost always resulted in a considerable decrease of average LF0. Only in two cases, for the two signals with multiple mode changes during highest SLN activation, LF0 increased during a detected mode change. In Fig 3, an example for a slow increase of LF0 during no SLN stimulation is shown. Fig 4 depicts an example for a typical mode change as we observed them in our data. Plots for all activation level combinations with sufficient phonation length are collected in S3 File.

For all data sets, DR increased over time with increasing flow. Mode changes also often resulted in a more sudden and noticeable increase of DR, i.e., GAW oscillation amplitude (see Fig 4). To compare DR and LF0 before and after the mode changes, median values of up to 10 cycles immediately before and after the mode change were calculated. In Table 4 mean values and standard deviations of these values before and after the mode changes based on SLN level are given.

**Table 4:** Median values for (up to) 10 cycles before and after a mode change were calculated for LF0 and DR. In this table the mean values and standard deviations for these values based on SLN level are given.

SLN level	LF0 before	LF0 after	DR before	DR after
0	-	-	-	-
1	350 / 17	205 / 7	2372 / 955	3412 / 1242
2	348 / 21	212 / 10	1963 / 736	3078 / 1135
3	396 / 26	236 / 8	3002 / 488	3933 / 581
4	402 / 0*	250 / 0*	2746 / 0*	3888 / 0*

* only signals with single mode changes were included i.e. only one signal for this SLN level

The parameter PHA in general reflected asymmetric stimulation as shown in Fig 2. Further, it was also in agreement with subjective asymmetry ratings made on the same video data in previous work [19]. However, in some cases the glottis showed oscillatory behavior that artificially decreased PHA (see Shortcomings).

**Fig 5:**
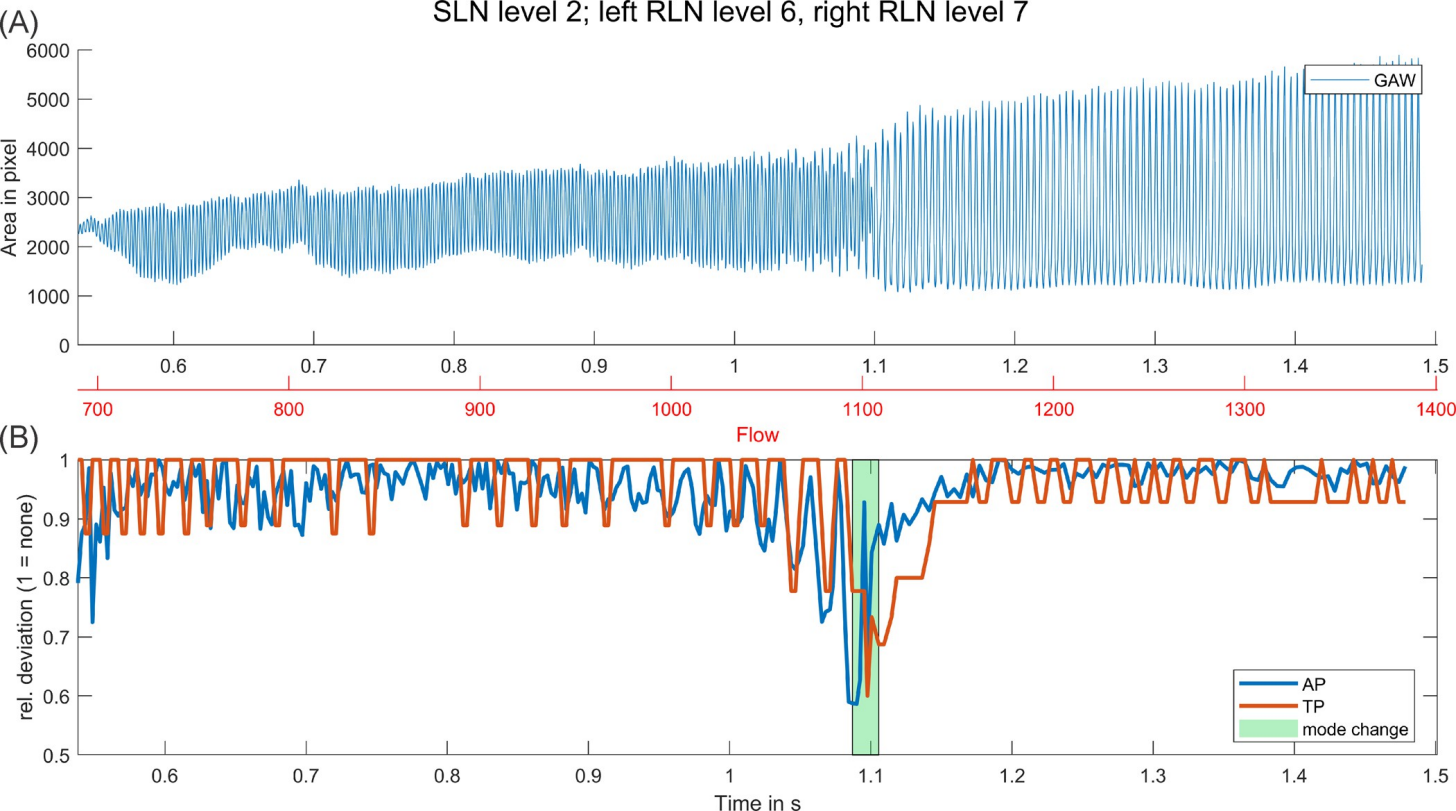
Perturbation before and during a mode change. (A) GAW over period of stimulation with increasing flow. (B) AP and TP over same period with marked mode change at 1.1 s.

AP and TP generally stayed relatively stable with increasing flow with two exceptions. In some signals higher perturbation (i.e. lower TP and / or AP) was measured in the earliest section of the signal, close to onset. Further, perturbation was increased considerably during, and in some cases right before, a mode change, as depicted in Fig 5. Also, whilst perturbation in general did not increase noticeably with increasing SLN level, perturbed sections before and after the actual frequency drop of mode changes increased.

**Fig 6:**
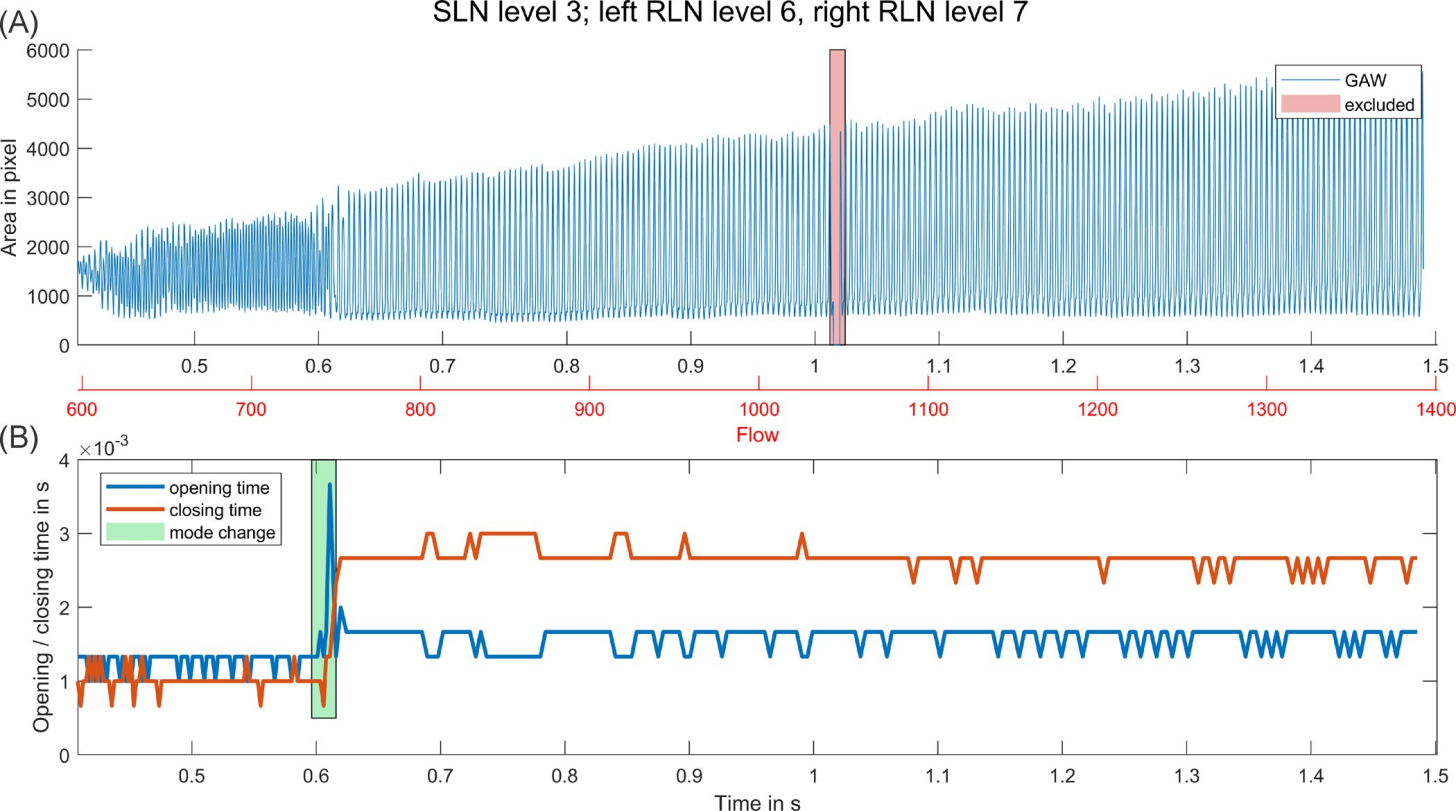
Perturbation before and during a mode change. (A) GAW over period of stimulation with increasing flow. (B) AP and TP over same period with marked mode change at 0.6 s.

OD and CD stayed mostly stable with increasing flow with either (a) OD and CD being of about equal length, or (b) CD being noticeably longer than OD. Only in a minority of cases OD was longer than CD. However, if a mode change occurred this always resulted in an increased CD afterwards, but no similarly strong increase in OD. Therefore, mode changes induced a shift from oscillation patterns with about equal OD and CD to oscillation patterns with significantly increased CD (see Fig 6).

**Fig 7:**
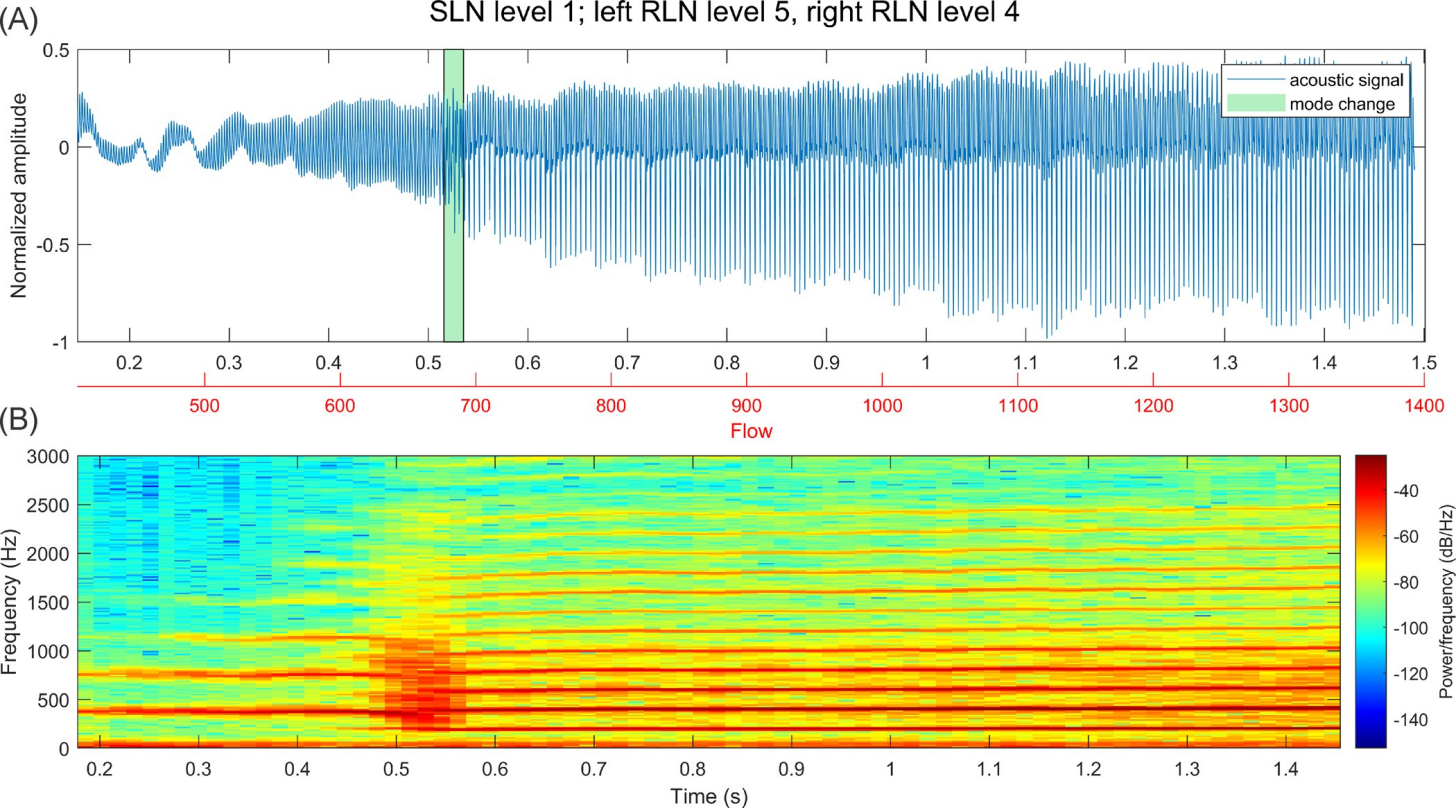
(A) Acoustic signal over period of stimulation with increasing flow and marked mode change (detected based on GAW LF0). (B) Spectrogram over same time period (4096 window length, 80% overlap).

In Fig 7, the acoustic signal and spectrogram for the same activation combination as depicted in Fig 4 is shown. As expected, the acoustic F0 follows the GAW F0. After the mode change, the dominance of harmonics but also spectral noise is increased. This effect was observable for all signals with mode changes, albeit not always similarly pronounced. Further, in some signals, a short disturbed section similar to the mode change section in Fig 7 (but less distinctive) could be observed, but no LF0 drop happened.

## IV. Discussion

For all five sets, the minimum amount of 0.25s of phonation achieved for analysis was more likely with increasing RLN stimulation. This is expected, as the RLNs activate all laryngeal adductor muscles to close the glottis and set up the phonatory position [8, 27], and hence facilitate phonation. Also, as the SLN stimulation level increased the duration of phonation decreased, as phonation onset occurred later during airflow ramp. As a higher SLN activation results in a higher vocal fold tension [8, 28], an increasing amount of energy (subglottal pressure) was required to initiate oscillation. With higher levels of SLN stimulation, higher corresponding levels of RLN stimulation were also required to reach conditions resulting in phonation.

The increase of DR over time with increasing flow for all stimulation combinations, and the good agreement between PHA and asymmetric stimulation were expected and serve as evidence that the input parameters were implemented, as intended. Put another way, higher flow led to stronger oscillation, and asymmetric stimulation led to asymmetric vibration, respectively.

### A. Parameter analysis

Mode changes were only observed if SLN stimulation was present, with the average LF0 being lower in the case of no SLN stimulation. As expected, with increasing SLN stimulation, LF0 also increased [29]. For the highest level of SLN stimulation at close to maximum RLN stimulation, oscillation patterns became sometimes chaotic, which resulted in multiple detected mode changes per signal in two cases but distinctly less mode changes overall for the highest SLN level. For chaotic sections the number and position of detected mode changes also becomes less reliable. The chaotic sections may be interpreted as an artifact of the very high stimulation, as the oscillatory system became unstable. However, it is also noteworthy that the overall lower number of mode changes (with only one “regular” mode change) principally agrees with the findings of Zhang 2018 whose model predicted more stable phonation with high transverse stiffness i.e. high CT activation [13]. Therefore, it may be that there is not only a minimum tension required to facilitate mode changes (increasing LF0 to allow frequency drops to happen in the first place), but also a maximum level of tension beyond which these sudden changes are impeded.

All observed mode changes besides the changes in the chaotic sections only resulted in a drop of LF0, never in an increase. At first this seems counterintuitive as with rising flow for conditions without SLN activation also LF0 increased (see Fig 3). However, the SLN activation leads to a distinctively higher LF0 already at onset. The change to a lower LF0 during a mode change with a simultaneous increase in dynamic range may hence reflect a change to a more stable state of the oscillatory system that allows for an easier passage of the rising airflow. In contrast, an even further increase of LF0 would rather hinder the passing of airflow.

Comparing LF0 and DR before and after mode changes shows only very little variation in LF0 for the different SLN levels (small standard deviations), which may suggest that certain frequencies are more stable during phonation. DR varies more but increases considerably during the mode changes (see also GAW DR increase in Figs 4–6(A)). With this increase in DR and a longer open time due to the lower LF0, the condition after the mode changes seems to be better suited for the increasing flow.

Mode changes happened more frequently for higher RLN stimulation and, interestingly, were also more common for symmetric stimulation. This behavior may be explainable as follows:

RLN activation brings the vocal folds closer together and therefore narrows the space between them. Similarly, symmetric activation leads to a better alignment of the folds and hence a smaller glottal space than asymmetric activation at similar activation levels. With increasing pressure and increasing airflow through the glottis (due to the narrowing glottal space), the system becomes unstable. In these cases, a mode change to a slower but wider oscillation occurs. In contrast, for conditions with less tightly aligned vocal folds, the glottal space is too large to allow for a sufficiently strong airflow to induce a mode change. It has been found that a light degree of phase asymmetry is found in a significant portion of normophonic speakers [30], raising the possibility that slight asymmetries in vibration may actually stabilize phonation, especially if increased intensity is desired.

As indicated by decreasing AP and TP slightly before a mode change (i.e. increasing perturbation), mode changes occur as the current configuration of the oscillatory system becomes unstable. Noteworthy, AP and TP often start decreasing before the actual drop in median F0 starts. With increasing SLN stimulation, the unstable intervals around the LF0 drop also increase, showing the increasing tension of the system. Further, it may be that the actual instability does not start earlier than the modal transition, but only that the existing instability becomes more visible.

As OT and CT are measured based on the cycle minima, they are affected if the cycle minimum is “noisy”, therefore “jumps” in CT and OT sometimes occur between neighboring cycles. However, by looking at the overall picture, the trend of increasing CT after mode changes is unambiguous. In this regard the observed mode changes are similar to changes between falsetto to chest register, which also result in an increased closing duration [31]. In contrast to AP and TP, no gradual change of CT and OT is noticeable right before or after the frequency drop.

**Fig 8:**
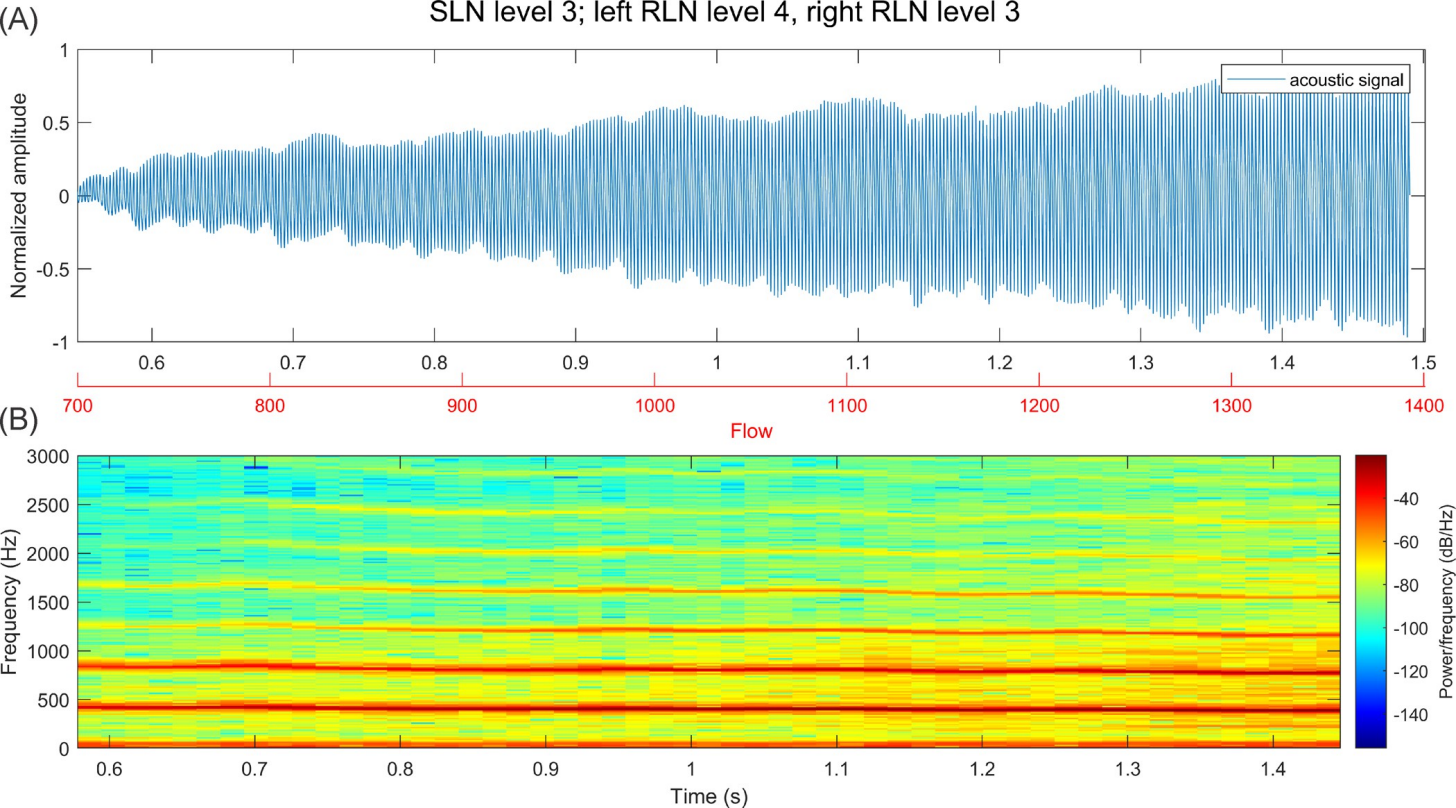
(A) Acoustic signal over period of stimulation with increasing flow (B) Spectrogram over same time period (4096 window length, 80% overlap).

With increasing flow, harmonics become slightly more prominent in the acoustic signal. Immediately after a mode change, harmonics also become more dominant in higher frequencies. However, no clear change in the acoustic spectra of the different activation combinations is identifiable before the mode change happens. Apart from the slight increase in perturbation in immediate vicinity of the LF0 drop, similarly no distinct pattern appeared in the GAW which differentiated signals with and without mode changes. In Fig 8, an example of an activation combination with high LF0 but no mode change is given (acoustic signal and spectrum), which looks very similar to the signal Fig 7 before the mode change.

As indicated before, it should be noted that essentially all the “regular” mode changes documented in this study show many similarities with the falsetto-to-chest register transition in the human singing voice. That, in the mode prior to each transition, the following is observed: (a) a relatively high fundamental frequency (usually around 400 Hz), (b) relatively weak harmonics, and (c) a roughly equal OD and CD. These are characteristics generally associated with the falsetto register in the human singing voice [9–12]. On the other hand, in the mode subsequent to each transition, the following is observed: (a) a relatively low fundamental frequency (usually around 200 Hz,), (b) relatively strong harmonics, (c) a CD which is much longer than the OD. These are characteristics generally associated with the chest register in the human singing voice [10–12]. Thus, the spontaneous jumps we have observed in the study appear to be consistent with the falsetto-to-chest register transition frequently observed in the human singing voice.

In regards to the relationship of the current findings to certain neuromuscular voice disorders, the experimental paradigm resembles different states of neurogenic vocal fold paresis. Vocal paresis is one of the most common conditions encountered in clinical practice and patients can present with complaints of reduced vocal amplitude, vocal range, or vocal quality. These symptoms stem from abnormalities in phonatory posture and control of vibration. However, glottal opening phase asymmetry is a common finding on videostroboscopic examination of the larynx and is present in dysphonic as well as normophonic subjects. Thus, the clinical significance of such finding remains controversial (Haben et al.) [32]. Our findings support the notion that mild degrees of asymmetry might be beneficial for improved stability of the larynx to rising aerodynamic load. However, this concept requires further refinement and investigation.

### B. Shortcomings

For feasibility reasons we first desired to do a comprehensive, systematic study on a single subject. Given the success of this study, our next step will be to apply this study to a larger sample size. Nevertheless it has to be stated that all findings in this work result from measures taken from a single dog, which limits their generalizability. However, the findings did show a consistent picture over all 320 activation combinations and similar patterns were observed for related experiments in our laboratory, although they are not published yet.

As the experimental assembly and data evaluation procedure of this experiment is rather complex, an exact replication may be difficult. To ensure replicability, we added all code we used for data evaluation in the supporting information and only used already established procedures that are described in more detail in the cited sources [19, 24].

Albeit PHA reflected the general asymmetry of vocal folds oscillation well, it may not be useful for quantifying the *exact degree* of asymmetry. The reason for this is, that PHA measures the distance between the maximum of the left half and the maximum of the right half of the glottal area during one oscillation cycle. In some cases, a certain oscillation behavior occurred during which the glottis opened with a phase shift not only in the left-right direction, but also in the anterior-posterior direction. This anterior-posterior phase shift “blurred” the actual position of the left and right maximum openings, and hence artificially altered (i.e., increased or decreased) the PHA. As our data shows, this effect was not sufficiently strong to hide the general left-right asymmetry, but it impeded the utility of the PHA in precisely quantifying left-right phase asymmetry.

We assessed acoustic spectra and general glottis oscillation in this work but did not investigate oscillatory behavior in more detail using e.g. phonovibrograms [33]. In part, this was because the damaged sections in most of the videos led to GAT not being able to scale the phonovibrograms it calculates from the raw data correctly. It may be that a deeper look into the glottis contour and local features reveals some subtle changes in the oscillation that precede an actual mode change happening.

## V. Conclusion

In this study, we explored mode changes occurring in a canine voice during symmetric and asymmetric left/right RLN stimulation at multiple levels of symmetric SLN stimulation. We assessed the acoustic signal, acoustic spectra and the GAW. To objectively investigate oscillatory behavior, seven different GAW based parameters were calculated. All algorithms used to detect the mode changes and calculate the different investigated features are included in this work to secure reproducibility.

Mode changes only occurred when the SLNs were stimulated and were more frequent in case of more symmetric and stronger RLN stimulation. A slight increase in aperiodicity and amplitude perturbation usually slightly preceded the F0 drop that happened during a mode change. However, apart from this no signal-based feature was found, that differentiated signals with and without mode changes. Significantly, the spontaneous mode changes documented in this study show many similarities with the falsetto-to-chest register transition in the human singing voice.

With this work, fundamental relations between nerve activation and oscillatory stability were explored. Its findings serve as one of the many pieces in the puzzle which ultimately yields a clear, consistent picture of voice, voice production, voice modulation, and phonatory stability of neuromuscular activation states in an *in vivo* larynx.

## Supporting information

S1 File Matlab functions for cycle detection, data polishing and parameter calculation. (M)

S2 File Matlab function for mode change detection based on LF0 change. (M)

S3 File Collection of all 732 parameter plots for 183 activation combinations with a minimum of phonation for the overly interested reader. All activation plot figures are available online at https://doi.org/10.5281/zenodo.5832024.
(TXT)
